# Proteins DotY and DotZ modulate the dynamics and localization of the type IVB coupling complex of *Legionella pneumophila*

**DOI:** 10.1111/mmi.14847

**Published:** 2021-12-06

**Authors:** Kevin Macé, Amit Meir, Natalya Lukoyanova, Luying Liu, David Chetrit, Manuela K. Hospenthal, Craig R. Roy, Gabriel Waksman

**Affiliations:** ^1^ Institute of Structural and Molecular Biology Birkbeck and UCL London UK; ^2^ Boyer Center for Molecular Medicine Department of Microbial Pathogenesis Yale University New Haven Connecticut USA; ^3^ Institute of Structural and Molecular Biology University College London London UK; ^4^ Present address: Institute of Molecular Biology and Biophysics Department of Biology ETH Zürich Otto‐Stern‐Weg 5 Zürich 8093 Switzerland

**Keywords:** coupling complex, effector recruitment, *Legionella pneumophila*, recruitment platform, type 4 secretion system

## Abstract

*Legionella pneumophila* is an opportunistic pathogen infecting alveolar macrophages and protozoa species. *Legionella* utilizes a Type IV Secretion System (T4SS) to translocate over 300 effector proteins into its host cell. In a recent study, we have isolated and solved the cryo‐EM structure of the Type IV Coupling Complex (T4CC), a large cytoplasmic determinant associated with the inner membrane that recruits effector proteins for delivery to the T4SS for translocation. The T4CC is composed of a DotLMNYZ hetero‐pentameric core from which the flexible IcmSW module flexibly protrudes. The DotY and DotZ proteins were newly reported members of this complex and their role remained elusive. In this study, we observed the effect of deleting DotY and DotZ on T4CC stability and localization. Furthermore, we found these two proteins are co‐dependent, whereby the deletion of DotY resulted in DotZ absence from the coupling complex, and vice versa. Additional cryo‐EM data analysis revealed the dynamic movement of the IcmSW module is modified by the DotY/Z proteins. We therefore determined the likely function of DotY and DotZ and revealed their importance on T4CC function.

## Graphical abstract

*Legionella pneumophila* utilizes the type 4 secretion system (T4SS) to translocate over 300 effector proteins into its host cell. These are recruited by a multiprotein complex, the type 4 coupling complex (T4CC), which is essential for effector binding and delivery to the T4SS. Two newly discovered proteins, DotY and DotZ, previously shown to be part of the T4CC, are here further characterized, leading to novel findings regarding T4CC dynamics, assembly, and cellular localization.

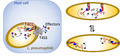

## 1 INTRODUCTION

*Legionella pneumophila*, the causative agent of *Legionnaire*'s disease (Brenner et al., 1979), is an opportunistic human pathogen which evolved from infecting protozoan hosts to infecting human alveolar macrophages as well (Swart et al., 2018). The bacterium translocates over 300 effector proteins into the host cytosol, where they hijack cell functions in order to create a specialized organelle, called the *Legionella* containing vacuole (Qiu & Luo, 2017), that supports intracellular replication. Recent studies have demonstrated the adaptability of the secreted effector subset depending on the infected host (Park et al., 2020). To translocate these effectors, *L. pneumophila* uses a specialized secretion system, called the Dot/Icm Type IVB Secretion System (Li et al., 2019; Waksman, 2019) (T4BSS), encoded by 29 different *dot*/*icm* genes. The T4BSS is a complex nanomachine made up of several sub‐complexes, one of which, called the Type IV Coupling Complex (T4CC), has the primary function of recruiting effectors and delivering them to the trans‐membrane machinery for translocation into host cells. Integrated into the inner membrane, the T4CC is a multiprotein effector recruitment platform comprising different effector binding sites and an AAA+ ATPase called DotL.

In a previous study (Meir et al., 2020), using cryo‐electron microscopy (cryo‐EM), we determined the structure of the T4CC of *L. pneumophila*. The structure revealed that the T4CC is made of two parts linked by the C‐terminal tail of DotL (DotL_Cter_): the hetero‐pentameric core composed of the DotL ATPase domain, DotM, DotN, DotY, and DotZ (referred to as “DotLMNYZ core”), and the flexible IcmSW module composed of IcmS and IcmW (Kwak et al., 2017; Meir et al., 2020; Vincent et al., 2012). The DotY and DotZ proteins had not been reported before and the study showed that DotY/Z play a significant role in effectors translocation but are not essential. DotL belongs to the VirD4‐family of AAA+ ATPases. Although the DotL complex was purified as a monomer, this family of ATPases typically function as hexamers (Gomis‐Ruth & Coll, 2001; Gomis‐Ruth et al., 2002); thus, we suggested that the T4CC assembles into a 1.6 MDa starfish‐shaped hexamer. Finally, the T4CC contains at least two binding sites for recruitment of two different classes of effectors: one on DotM, proximal to a cavity at the center of the hetero‐pentameric core (Meir et al., 2018), and the second on the IcmSW module (Cambronne & Roy, 2007; Sutherland et al., 2012). Because DotL_Cter_‐bound IcmSW is flexibly linked to the DotLMNYZ core, we probed the trajectory of the IcmSW module relative to the core using cryo‐EM and suggested that its trajectory is consistent with bringing IcmSW‐bound effectors to the central channel of the core hexamer (Meir et al., 2020).

Here, we further investigate the function of DotY and DotZ proteins. First, we obtained a near‐atomic resolution cryo‐EM map that includes the middle domain of DotY previously missing. After determining that DotY and DotZ are co‐dependent for assembly into the T4CC, we resolved the cryo‐EM structure of the T4CC in absence of DotY and DotZ proteins. Further analysis reveals that the trajectory of the IcmSW module is modified by DotY/Z, thereby suggesting the likely function of these proteins. Finally, we determined by in vivo fluorescence that DotY and DotZ have an influence on the polarity of the T4CC.

## 2 RESULTS AND DISCUSSION

### 2.1 DotY and DotZ sequence conservation, binding co‐dependence, and cryo‐EM structure of the DotLMN complex

**FIGURE 1:**
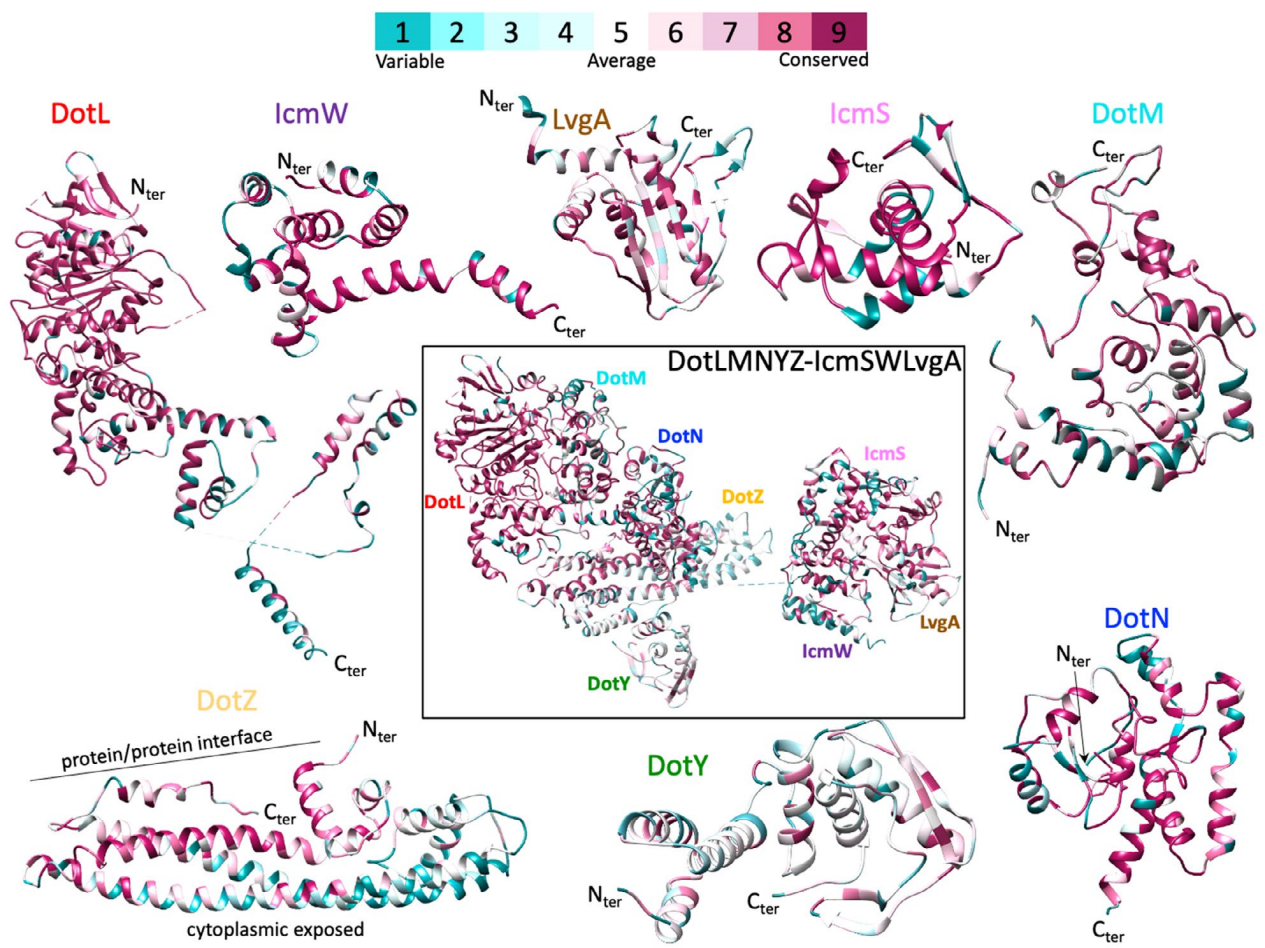
Residue conservation analysis. All proteins of the complex are shown in ribbon and colored by residue conservation, blue (variable) to magenta (conserved) as indicated in the color chart at the top. The sequence conservation was obtained from 39 *Legionella* species using ConSurf (Ashkenazy et al., 2016) (see Experimental Procedures for details)

We first analyzed the evolutionary history of DotY and DotZ and found that they are unique to the *Legionella* genus. Analysis of the sequence of DotY and DotZ showed that the two proteins are the least conserved components of the T4CC with 30%–50% conservation amongst *Legionella* species compared with 85%–90% for DotL (Figure 1). For DotZ, residues at the interface with other T4CC component are conserved, while residues facing the cytoplasm are not. For DotY, most residues are not conserved. DALI (Holm, 2020) analysis shows that DotY and DotZ do not belong to any structural or functional family. The facts that DotY and DotZ are not essential for secretion, unique to the *Legionella* genus, located at the periphery of the complex, do not belong to any known structural and functional families, and poorly conserved suggest that DotY and DotZ have only recently been evolved to play a part in type IV secretion in *Legionella*.

**FIGURE 2:**
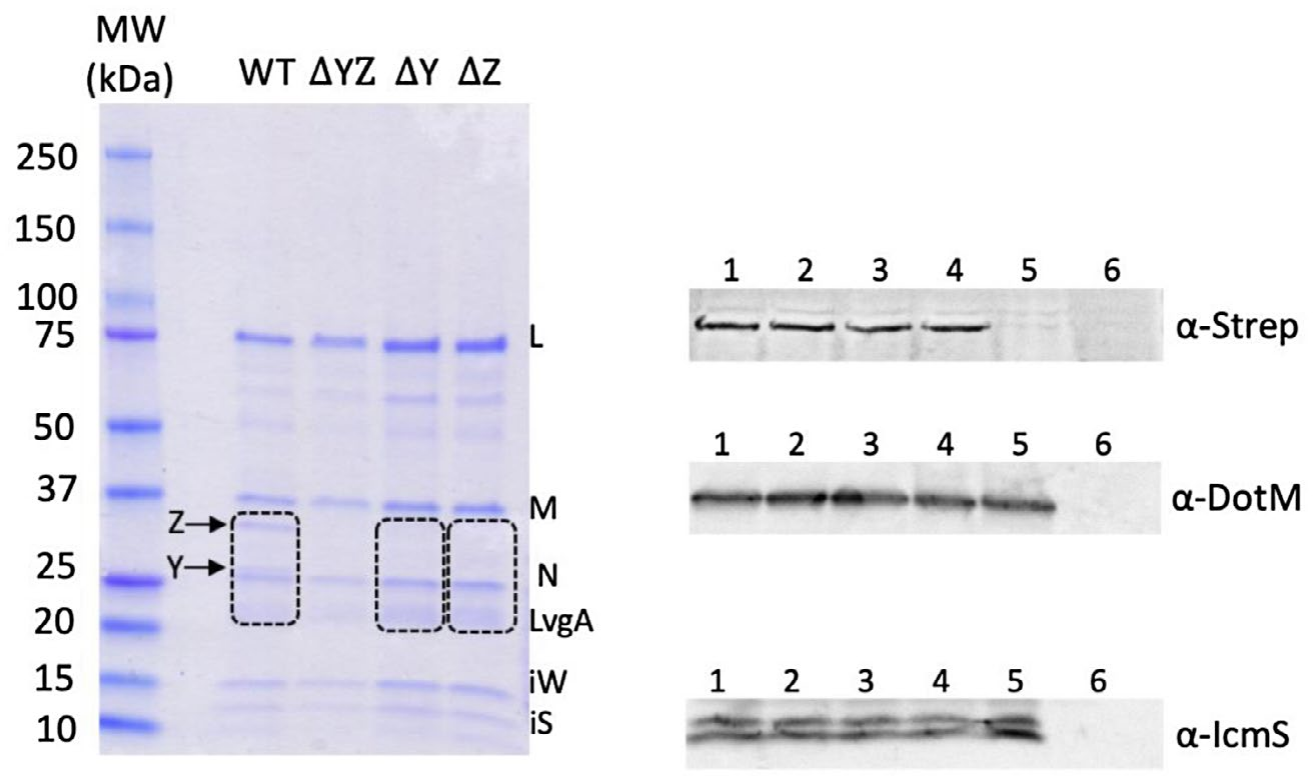
DotY/DotZ co‐dependence. (a) SDS–PAGE analysis of the purified T4CC from background strain Lp01 DotL_strep_. From left to right: MW, molecular weight markers; WT, T4CC_WT_ purified from Lp01 DotL_strep_ strain; ΔYZ, T4CC_ΔdotYZ_ purified from the *ΔdotYZ* strain; ΔY, T4CC_ΔdotY_ purified from the *ΔdotY* strain; ΔZ, T4CC_ΔdotZ_ purified from the *ΔdotZ* strain. Dashed line boxes indicate the portions of gel (as described in Experimental Procedures) used for mass spectrometry analysis, the results of which are reported in Table S2. Molecular weights for lane MW are provided (kDa). Y, Z, L, M, N, IS, and IW indicate DotY, DotZ, DotL, DotM, DotN, IcmS, and IcmW, respectively. DotY migrates just above DotN. SDS–PAGE analysis of the complex was routinely carried out after each preparation (at least two times for each strain) and yielded the same result. (b) Western blot of SDS–PAGE gel using anti‐StrepII (upper panel), anti‐DotM (middle panel), and anti‐IcmS antibodies indicating DotL, DotM, and IcmS levels remain the same in all wild‐type and knockout mutants. 1‐ T4CC_WT_, 2‐ T4CC_ΔdotY_, 3‐ T4CC_ΔdotZ_, 4‐ T4CC_ΔdotYZ_, 5‐ Lp01, 6‐ Lp01_ΔT4SS_

To investigate DotY and DotZ stability and function in the T4CC, we deleted each gene separately in *L. pneumophila* Lp01 and Lp02 DotL_Strep_ backgrounds (strains termed thereafter “Δ*dotY*” and “Δ*dotZ*”), and also generated a strain with both genes deleted (strain termed thereafter “Δ*dotYZ*”) (see Experimental Procedures and Table S1). Purification of the T4CC (using the Strep‐tag at the C‐terminus of DotL) from the Δ*dotY* or Δ*dotZ* strain resulted in the absence of both the DotY and DotZ proteins, similar to the Δ*dotYZ* strain (Figure 2a; Figure S1). The absence of these proteins within these various T4CC complexes was confirmed by mass spectrometry (See Experimental Procedures and Table S2). Thus, the lack of one protein results in the other one being unable to assemble with remaining T4CC components. In the T4CC structure, DotY interacts exclusively with DotZ, which would explain why DotY would depend on DotZ to co‐purify with the other T4CC components. On the other hand, the dependence of the DotZ protein on DotY interaction with T4CC components is unexpected, because DotZ makes intensive interactions with DotL_Cter_, DotM, and DotN. One potential explanation for this observation is that DotY stabilizes DotZ prior to assembly, allowing it to assume a conformation conducive to association with other T4CC components. Finally, using immunoblot analysis of DotL_strep_, DotM, and IcmS in the various T4CC complexes produced in the three deletion strains and the nondeleted one (referred to for clarity as “wild‐type” or “WT” even if it contains a Strep‐tag at the C‐terminus of DotL), we find that the levels of DotL, DotM, and IcmS remain the same in all strains despite the absence of DotY or DotZ or both (Figure 2b). This result demonstrates that DotY/Z does not influence the stability of the T4CC main components. We propose that DotY and DotZ might be co‐dependent on each other, suggesting they might act together as a module, similarly to other T4SS components that appear to function in pairs, such as IcmSW (Cambronne & Roy, 2007), IcmRQ (Raychaudhury et al., 2009), and DotIJ (Kuroda et al., 2015).

**FIGURE 3:**
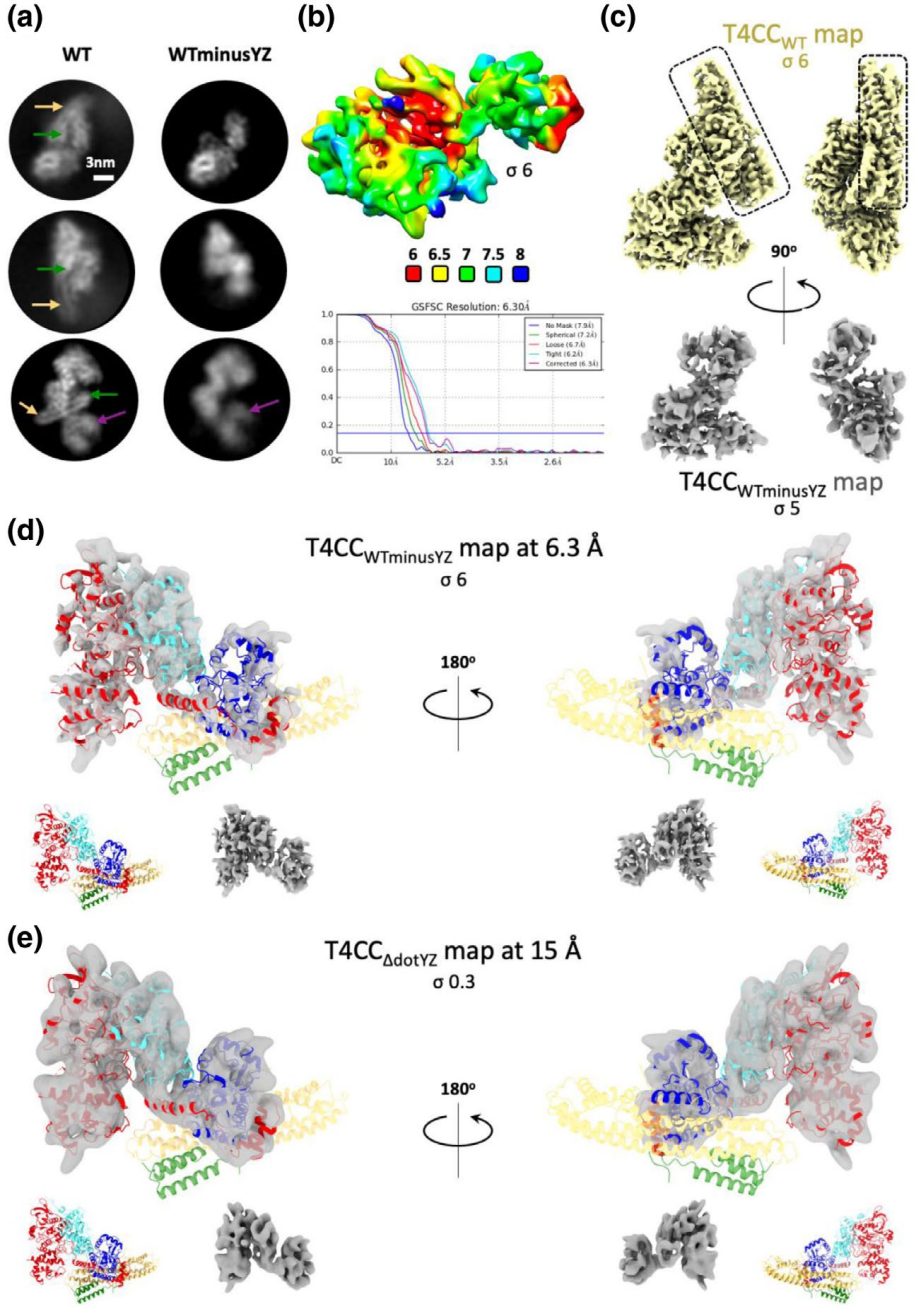
Superposition and comparison of the maps and structures of the T4CC with or without DotY and DotZ. (a) Example of 2D classes with or without DotY/Z (labeled WT and WTminusYZ, respectively) from the T4CC_WT_ dataset collected previously (Meir et al., 2020). Arrows in green, yellow and purple indicate the position of DotY, DotZ, and IcmSW, respectively. (b) Local resolution and FSC plot for the T4CC_WTminusYZ_ map. Local resolution was calculated using CRYOSPARC (FSC cut‐off 0.5) and colored as indicated in the scale below the map. The FSC plots is between two independently refined half‐maps with no mask (blue), spherical mask (green), loose mask (red), tight mask (cyan), and corrected (purple). Cut‐off 0.143 (blue line) was used for resolution estimation. (c). Comparison of the core region (DotLMNYZ) of the T4CC_WT_ map obtained in our previous study (Meir et al., 2020) (yellow; EMD‐8623; labeled “T4CC_WT_ map”; upper panels) and the core region (DotLMN) of the 6.3 Å resolution T4CC_WTminusYZ_ map (labeled “T4CC_WTminusYZ_” [grey]; lower panels). Dashed boxes indicate the densities present in the T4CC_WT_ map but absent in the T4CC_ΔDotYZ_ map. (d) Core region (DotLMN) of the 6.3 Å resolution T4CC_WTminusYZ_ map obtained from T4CC_WT_ particles where DotY and DotZ were missing (see Experimental Procedures). (e) DotLMN core region of the T4CC_ΔDotYZ_ map solved at 15 Å resolution (see Experimental Procedures). In panels d and e, the upper panels show two views of the superposition of the map and the DotLMNYZ core structure shown in ribbon representation color‐coded red, cyan, blue, orange yellow, green for DotL, DotM, DotN, DotZ, DotY_NTD_, respectively. The lower panels show the DotLMNYZ core structure and the map shown above but side by side. As can be seen, the core structures are identical except for the absence of DotY and DotZ. σ levels for all maps are indicated

We next solved the cryo‐EM structure of the T4CC in the absence of DotY and DotZ at a resolution of 6.3 Å (Figure 3a,b; Table S3) by taking advantage of the sample heterogenicity that we observed in the dataset we collected previously on the wild‐type T4CC (DotLMNYZ‐IcmSW; termed thereafter “T4CC_WT_”) (Meir et al., 2020). Indeed, in this dataset, a substantial proportion of particles do not have DotY and DotZ (Figure 3b) and therefore these particles are representative of the DotLMN–IcmSW complex where DotY and DotZ are missing (termed thereafter T4CC_WTminusYZ_). When the resulting T4CC_WTminusYZ_ map is compared with that obtained previously for T4CC_WT_, no significant difference is observed, except for the absence of DotY and DotZ (Figure 3c,d). We confirmed this by collecting a small cryo‐EM dataset on the T4CC purified from the Δ*dotYZ* strain, which yielded a map at a low resolution of 15 Å (Figure 3e; named “T4CC_Δ_ *_dotYZ_*” map; Table S3), which shows no difference compared with the 6.3 Å resolution T4CC_WTminusYZ_ map described above (Figure 3d,e). Thus, the absence of DotY and DotZ proteins does not affect T4CC core formation, confirming the conclusion of the immunoblot experiment that shows DotL unaffected by the absence of DotY or DotZ or both. All these results are consistent with our previous study showing that DotY and DotZ play a role in effector translocation but are not essential (Meir et al., 2020).

### 2.2 Additional information on the structure of DotY

**FIGURE 4:**
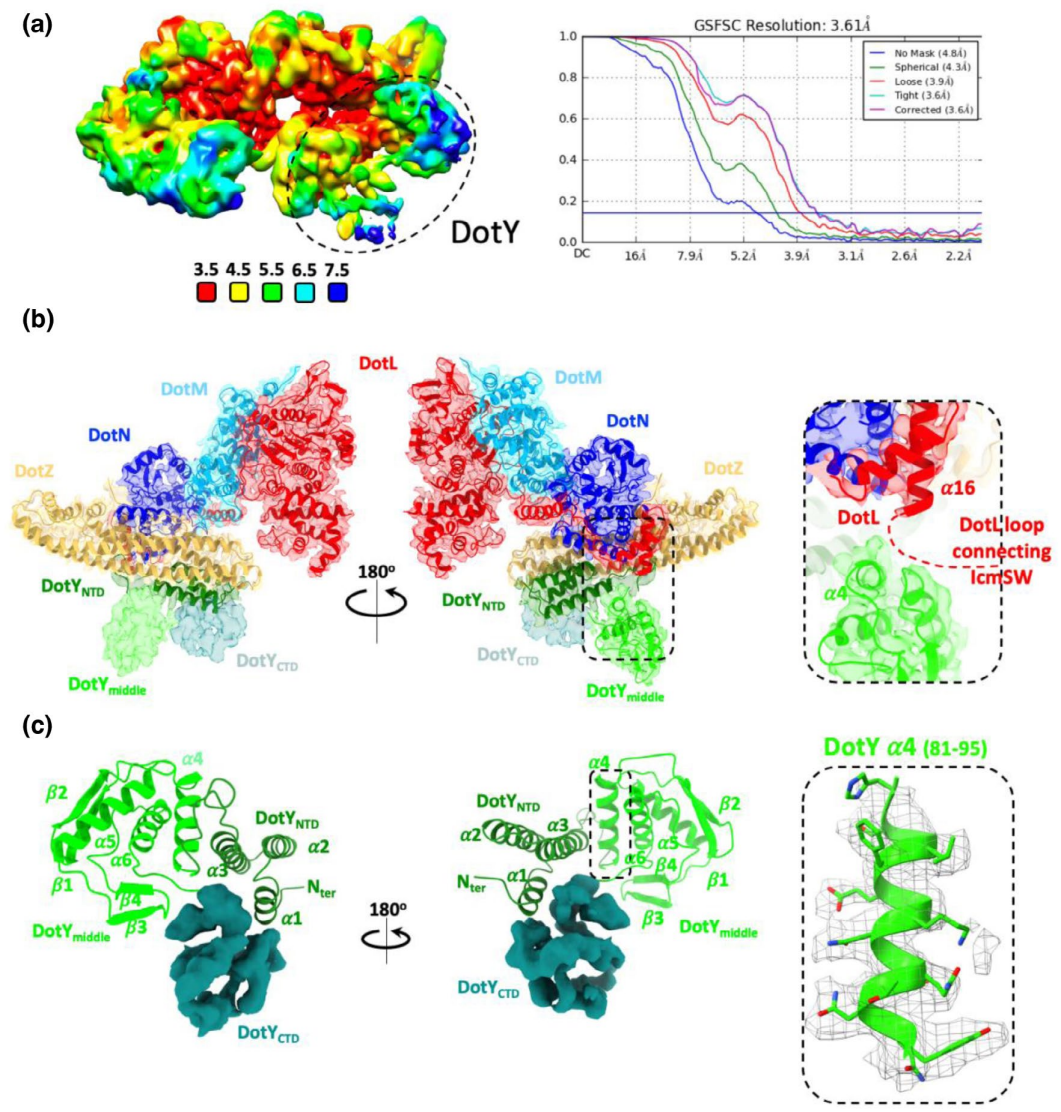
DotY cryo‐EM structure. (a) Local resolution and FSC plot for the “reprocessed T4CC_WT_” map used to complete the structure of DotY (the position of which is shown by a dash lined oval). Local resolution was calculated using CRYOSPARC and colored as indicated in the scale below the map. The FSC plots is between two independently refined half‐maps with no mask (blue), spherical mask (green), loose mask (red), tight mask (cyan), and corrected (purple). Cut‐off 0.143 (blue line) was used for resolution estimation. (b) The 3.61 Å resolution reprocessed T4CC_WT_ cryo‐EM map used to build a more complete model of DotY. The DotLMNYZ structure obtained previously (PDB 6SZ9) is shown fitted into the density in ribbon representation color‐coded as in Figure 3b. This map shows the extra density corresponding to the DotY_middle_ (pale green) and DotY_CTD_ (light blue) domains missing in the previous structure. Inset at right: zoom‐in view of a potential interaction between the DotY_middle_ domain and the C‐terminal tail of DotL connecting the T4CC core to the IcmSW module. (c) Structure of DotY. DotY_NTD_ (dark green) and DotY_middle_ (pale green) are shown in ribbon representation. The density for DotY_CTD_ is colored teal. Secondary structures are labeled, as well as the N‐terminus. Inset at right: electron density for α4 of DotY_middle_ between residues 78 and 106 showing clear side chain definition. The map shown in all panels is contoured at σ 7

One part of the DotLMNYZ core structure missing in our previous study was the structure formed by residues 78–230 of DotY (Meir et al., 2020). The residues prior to residue 78 form a three‐helix bundle that makes tight interactions with DotZ, hence their good definition in the electron density. However, the density for residues C‐terminal to residue 77 (residues 78–230) was not interpretable, likely due to greater flexibility, and therefore no model was built at the time. Here we have reprocessed the T4CC dataset collected previously to focus on this specific area and improved the map (termed “reprocessed T4CC_WT_ map” with an average resolution of 3.61 Å (Figure 4a,b; Table S3)). In this map, improved density resulted in a model that included side chains for residues 78–106. For residues 107–192, only the main chain could be assigned whereas no continuous density was obtained for the remaining sequence (residues 193–230) (Figure 4b,c). Nevertheless, this part of the structure (residues 193–230) forms a distinct globular density domain (Figure 4c). Thus, the DotY structure is defined by three domains: the N‐terminal domain (residues 1–77; DotY_NTD_), a middle domain (residues 78–192; DotY_middle_), and a C‐terminal domain (residues 193–230; DotY_CTD_). The DotY_middle_ structure was unknown. It consists of a 3‐helices core (α4‐6) flanked by 2 two‐stranded β‐sheet. DotY_NTD_ and DotY_middle_ interact through residues in α3 and α4, respectively (Figure 4c). There are no contact between DotY_middle_ and the rest of the T4CC components. α4 is also in close proximity to the DotL linker connecting the DotLMNYZ core complex to the IcmSW module (Figure 4b inset at right), an observation that will be further discussed in the next section. DotY_CTD_ is oriented toward the cytoplasm and does not interact with other proteins of the complex, hence its flexibility, and, as a result, the definition of the map in this region is poor.

### 2.3 DotY and DotZ modulate the trajectory of IcmSW

IcmSW is a module that binds effectors, some *via* the LvgA adaptor protein (Cambronne & Roy, 2007; Kim et al., 2020). The IcmSW module is bound to the very C‐terminus of DotL at the end of a long and flexible linker (residues 659–688) that projects the IcmSW module ≃40 Å away from the DotLMNYZ core. In our previous study (Meir et al., 2020), using cryo‐EM, we were able to gain some insight into the dynamics of the system and demonstrated that IcmSW moves along a defined trajectory that may facilitate delivery of IcmSW‐bound effectors to the central channel of the T4CC hexamer or to some other components of the T4BSS. We also hypothesized that the length and flexibility of that linker allow the IcmSW module to move over a larger volume, thereby affording the search of a wider volume of cell space (termed “search volume”), increasing the chance of a collision with an effector and therefore its binding.

**FIGURE 5:**
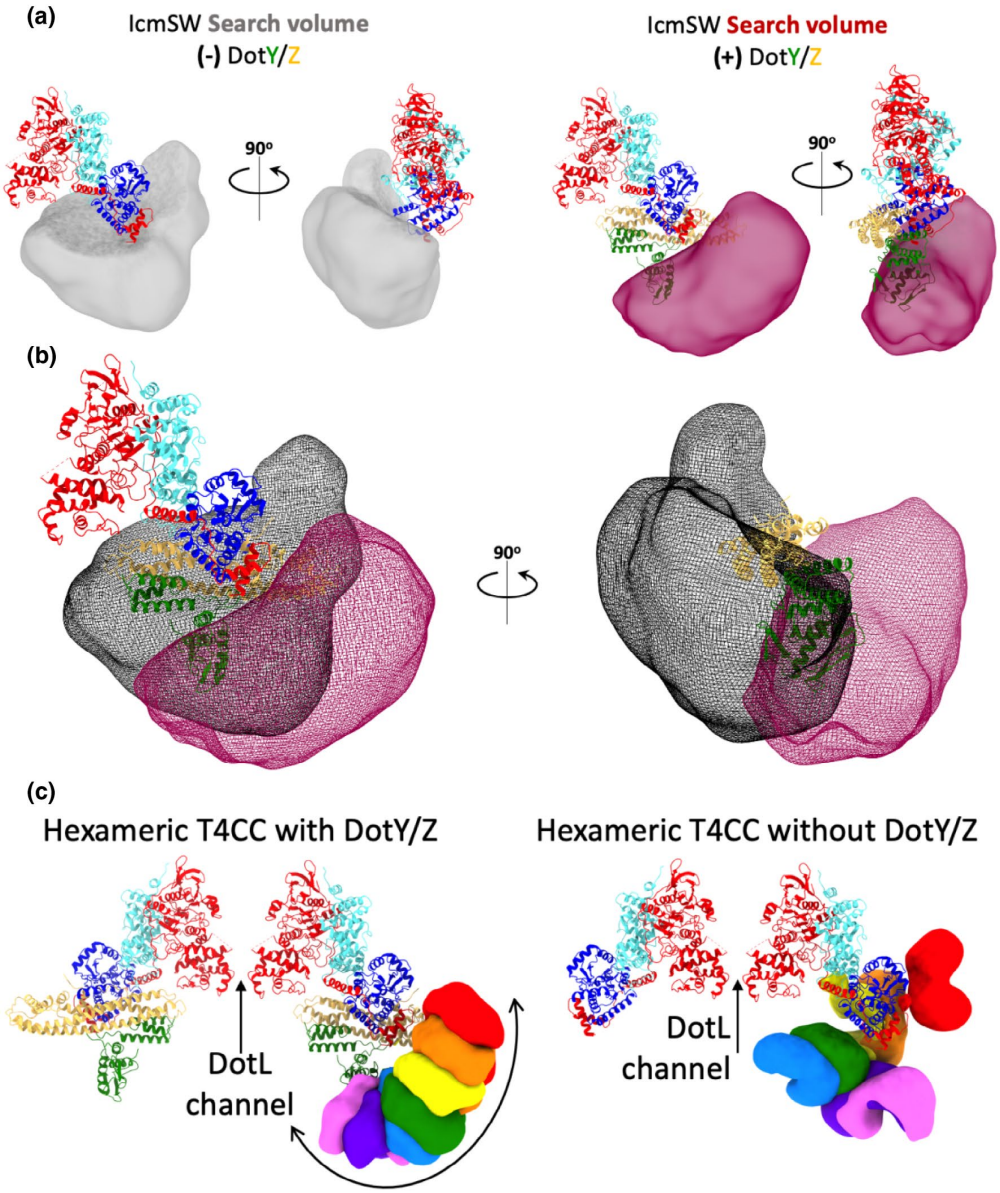
Analysis of IcmSW search volume in the presence or absence of DotYZ. In panel a, b, and c the T4CC core structure (either DotLMN or DotLMNYZ) is shown in ribbon representation color‐coded per proteins as in Figure 3. (a) Search volumes of IcmSW in the context of either the DotLMN core (in grey at left) or the DotLMNYZ core (in red at right). (b) Superposition of the IcmSW search volumes in the context of either the DotLMN core (in black) or the DotLMNYZ core (in red). The fitted model includes the newly determined DotY_middle_ domain, which, at right, is shown to overlap with the volume in black. (c) Trajectory of the IcmSW module illustrated by the superposition of the seven best resolution maps obtained for IcmSW color‐coded differently in the context of the DotLMNYZ core (left) or the DotLMN core (right)

Here, we asked whether DotY and DotZ play a role in defining the size, shape, and location of the search volume of IcmSW and its trajectory. To do so, we repeated the previous analysis but, this time, on the T4CC_WTminusYZ_ particles described above. Multiple maps were generated, each representative of a distinct orientation of the IcmSW module relative to the DotLMN core. All these positions were used to define the “search volume” as defined above, i.e. the volume within which the IcmSW moves. It is shown in a grey surface in Figure 5a, left panel. Also, from the results obtained in our previous study (Meir et al., 2020), the search volume of the IcmSW module in the context of the fully assembled T4CC_WT_ complex was derived (shown in red in Figure 5a, right panel). As can be seen, the volumes are similar in size (433.7 Å^3^ and 387.1 Å^3^, in the presence or absence of DotY/Z, respectively) but different in shape and location (see superposed volumes in Figure 5b). While the volume in red is regularly shaped, indicating motions restrained within a defined trajectory, the volume in black is not. Instead, without DotY and DotZ, the IcmSW module positions itself more randomly. As shown in Figure 5c, the motions of IcmSW in the context of the T4CC_WT_ complex follow a trajectory conducive to directing IcmSW‐bound effectors to a putative DotL channel. In the absence of DotY and DotZ, this is not the case: although some IcmSW positions are proximal to a DotL channel, many are not, and none define a trajectory. We also observe that some of the IcmSW positions are found where DotY and DotZ locate in the T4CC_WT_ complex. Thus, the absence of DotY and DotZ results in the modification of not only the shape but also the location of the search volume. Given the proximity of the DotY_middle_ domain and the C‐terminal tail of DotL (shown in Figure 4b in inset at right), it may not be surprising that, in the absence of DotY, the IcmSW module may occupy some of the vacant DotY/Z space. These observations lead us to suggest the following: (a) because the search volumes are very similar in size, we may conclude that DotY/Z do not play a role in affecting the likelihood of IcmSW colliding with an effector and binding it; (b) because IcmSW locate in random positions in the absence of DotY and DotZ, we may conclude that DotY and DotZ constrains IcmSW within the motion trajectory directing IcmSW‐bound effectors to a putative DotL channel. In their absence, effectors can still reach the channel, but likely less often than in their presence. Thus, these results suggest a role for DotY and DotZ: they optimize effector delivery by the effector‐capturing IcmSW module.

### 2.4 DotY and DotZ affect the T4CC cellular localization

**FIGURE 6:**
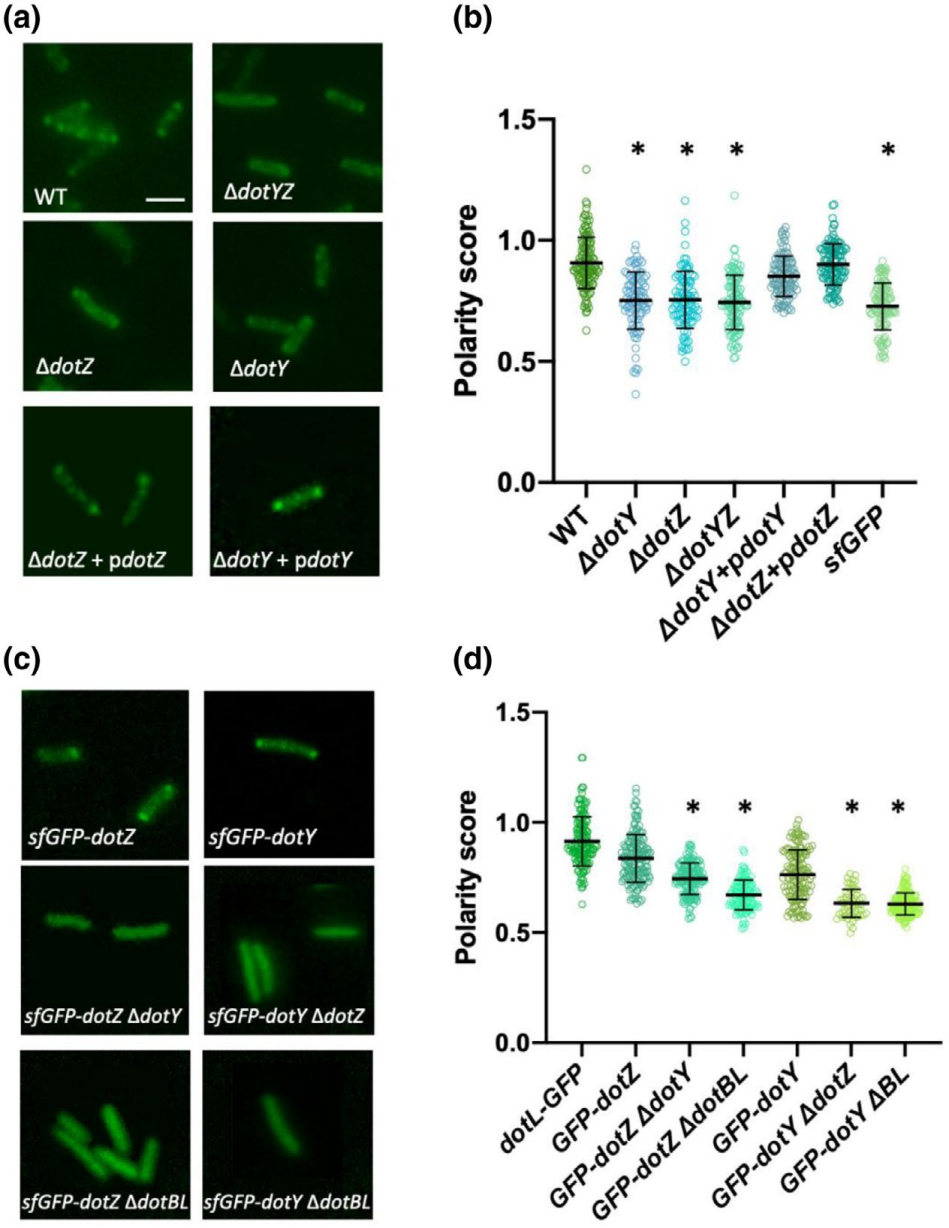
DotY/DotZ affect the T4CC polar localization in *Legionella pneumophila*. (a) Real‐time visualization with fluorescence light microscopy of DotL‐sfGFP localizes to *L. pneumophila* poles in Lp01 WT background strain; however, this localization is lost at the Δ*dotY*, Δ*dotZ*, and DKO background strains. This polarity is restored upon introduction of a DotY/DotZ copy on a plasmid. (b) The polar localization of the DotL‐sfGFP WT and mutant strains polarity is displayed as scattered dots. Median values ± *SD* of the polarity scores are presented. The significance was calculated compared with DotL‐sfGFP wild‐type. As a control, sfGFP alone was expressed from the end of *dotB* operon. Scale bar, 2 μm. Each experiment was conducted three times. *N*, the number of cells recorded was ≥100. All strains were compared with *dotL‐sfGFP* strain. *p* values of mutant strains in comparison with wild‐type were calculated by two‐tailed Student's *t* test. *p value <.001. (c) Real‐time visualization with fluorescence light microscopy of sfGFP‐DotY, sfGFP‐DotZ, sfGFP‐DotYΔDotZ, sfGFP‐DotZΔDotY, sfGFP‐DotYΔDotBDotL (ΔDotBL), and sfGFP‐DotZΔDotBL. Left, DotZ localizes to *L. pneumophila* poles in Lp01 wild‐type background strain; however, this localization is lost in the Δ*dotB/dotL* and Δ*dotY* background strains. Right, DotY weakly localizes to *L. pneumophila* poles in Lp01 WT background strain; however, this localization is lost at the Δ*dotL/dotB* and Δ*dotZ* background strains. (d) The polar localization of Dot/Icm fusion proteins in the wild‐type and mutant strains is displayed as scattered dots. Median values ± *SD* of the polarity scores are presented. The significance was calculated compared with wild‐type GFP fusion strain. Scale bar, 5 μm. Each experiment was conducted three times. *N*, the number of cells recorded was ≥100. Mutant strains were compared with their wild‐type GFP fusion strain, (e.g. *sfGFP‐dotYΔdotZ* to *sfGFP‐dotY* etc). *p* values of mutant strains in comparison with wild‐type were calculated by two‐tailed Student's *t* test. *p value <.001

The polarity of the T4BSS is important for function and *Legionella* virulence (Jeong et al., 2017). The T4CC has also been shown to localize to the poles of the bacterial cell (Chetrit et al., 2018; Vincent et al., 2012). To assess the effect of DotY and DotZ on T4CC polar localization we generated DotY/Z knockout strains with DotL fused with a superfolder Green Fluorescent Protein (sfGFP) (Experimental procedures and Figure S2). The Δ*dotY*, Δ*dotZ*, and Δ*dotYZ* deletions significantly reduced the DotL‐sfGFP polar localization, which could be restored to initial levels upon complementation of the deleted gene on a plasmid with its native promoter (Figure 6a,b). These results support a regulation of T4CC polar localization in which DotY and DotZ play a role.

We next examined the polar localization of DotY and DotZ themselves by introducing sfGFP to their N‐terminus. Both DotY and DotZ proteins have a polarity score close to DotL (Figure 6c,d). sfGFP‐DotZ in Δ*dotY* strain, or vice‐versa, resulted in abrogation of polarity, consistent with the previous results showing that DotY and DotZ are co‐dependent. Finally, in absence of DotB and DotL, proteins essential for the assembly and the polarity of T4CC, both DotY and DotZ did not exhibit polar localization (Figure 6c,d). These results show that DotY and DotZ proteins are not polar by themselves, but that their localization depends on the T4CC subcomplex. Overall, these assays support a regulation of T4CC localization at the cellular poles of *L. pneumophila* that depends on the fully assembled T4CC subcomplex, including DotY and DotZ.

Polar localization has been shown to be critical for a number of secretion machineries including T4SSs (Jeong et al., 2017). Given the high level of functional coupling between the T4CC and its cognate T4SS, it is not surprising that the T4CC locates at the cell poles. It has been shown that the Dot/Icm T4BSS is recruited at the poles through interactions with the cell division machinery, FtsZ or FtsI being a potential candidate (Jeong et al., 2017). Thus, localization of the T4CC to the pole could be achieved by either direct or indirect interactions of DotY–DotZ with a component of the T4SS or directly/indirectly through the Fts complex.

## 3 CONCLUSION

DotY and DotZ are two proteins we identified previously as nonessential components of the *Legionella* machinery that have an undefined role in the recruitment of effector proteins by the T4CC. Here, we show that DotY and DotZ are relatively recent additions to the evolutionary history of T4SS, with obvious homologs found only in the Legionellaceae family of bacteria. The DotY and DotZ proteins are functionally co‐dependent, and their main function is to optimize secretion by restricting (a) the delivery trajectory of the IcmSW module and (b) the localization of the machinery to the poles where the T4SS machinery resides. Our data highlight the particularities of a system that has long been evolved to accommodate many different hosts. It sheds new light on the mechanism of a system capable of orchestrating the secretion of hundreds of effectors and expands our understanding of the T4SS. Because of the functional diversity of its effectors, one of the largest in the bacterial world, *Legionella* has evolved a uniquely sophisticated recruitment platform. Further knowledge will lead to the design of engineered systems capable of selectively delivering therapeutic molecules and vaccines to animals and humans.

## 4 EXPERIMENTAL PROCEDURES

### 4.1 Bacterial strains and constructs

All strains and oligonucleotides used in this study are listed in Table S1.

For production of the knockout strains Δ*lpg0294* (Δ*dotY*) and Δ*lpg1549* (Δ*dotZ*) in the Lp01 and Lp02 DotL_strep_ backgrounds (previously described in Meir et al., 2020), the suicide pSR47S plasmids with corresponding knockout (previously described in Meir et al., 2020) were used to generate the strains. For production of the Δ*dotYZ* strain, after creation of DotL_strep_ Δ*dotY* strain, additional mutagenesis was performed with the Δ*dotZ* construct. All strains were verified by colony PCR.

For production of the GFP fusion strains GFP‐lpg0294 (GFP‐*dotY*) and GFP‐lpg1549 (GFP‐*dotZ*) in the Lp01 backgrounds, sfGFP (previously described in Chetrit et al., 2018) followed by a GAGGSSSGGGA (Gly‐Ala‐Gly‐Gly‐Ser‐Ser‐Ser‐Gly‐Gly‐Gly‐Ala‐) linker was cloned, using SLIC, into the suicide pSR47S plasmids upstream of the corresponding *dotY*/*dotZ* genes, allowing 1,000 bp at the 5′ and 3′ of the gene. For production of the knockout strains Δlpg0294 (Δ*dotY*) and Δlpg1549 (Δ*dotZ*) in the corresponding GFP fusion background strains, the suicide pSR47S plasmids with corresponding knockout (previously described in Meir et al., 2020) were used. For production of the Δ*dotB*/*dotL* strain, using the previously described Lp01 Δ*dotB* strain (Chetrit et al., 2018), additional knockout mutagenesis was performed with the Δ*dotL* construct. Then, the GFP‐fusion *dotY*–*dotZ* on the pSR47S construct were introduced. All strains were verified by colony PCR prior to fluorescent microscopy assays.

Lp01 DotL‐sfGFP strain (DotL_GFP_) has been previously described (Chetrit et al., 2018). For production of the knockout strains Δ*lpg0294* (Δ*dotY*) and Δ*lpg1549* (Δ*dotZ*) in the Lp01 DotL_GFP_ backgrounds, the suicide pSR47S plasmids with corresponding knockout (previously described in Meir et al., 2020) were used to generate the strains. For production of the Δ*dotYZ* strain, after creation of DotL_GFP_ Δ*dotY* strain, additional mutagenesis was performed with the Δ*dotZ* construct. All strains were verified by colony PCR.

For DotY–DotZ complementation assays, strains were transformed with *dotY* and *dotZ* cloned into the pJB1806 backbone with 200 bp upstream and downstream, so that native promotor is used for expression (as previously described in Meir et al., 2020).

### 4.2 Sample purification

*Legionella* cells were grown on charcoal yeast extract (CYE) plates or AYE medium containing appropriate antibiotics (100 μg/ml streptomycin and 10 μg/ml chloramphenicol) as previously described (Nagai et al., 2005).

For WT, Δ*dotZ*, Δ*dotY*, and Δ*dotYZ* T4CC complexes, purification was conducted as previously described. Briefly, 2‐days heavy patch cells were inoculated and grown for additional 26 hr in AYE medium and supplements to achieve a final OD_600_ of 3.2–3.6. Cells were harvested and resuspended in buffer LPA (40 mM Tris pH 8.0, 0.2 M NaCl, 2 mM EDTA, 20 mM MgSO_4_) and 0.5 M sucrose, 0.1 mg/ml lysozyme, DNAse I, and protease inhibitor (PI) (Roche). After rotation for 45 min at 4°C, cells were spun down and then re‐suspended in buffer LPB (50 mM Tris pH 8.0, 2 mM EDTA, 20 mM MgSO_4_, and PI), followed by three rounds of high pressure (40,000 psi) homogenization. After a short centrifugation to remove cell debris, samples spun for 2 hr at 167,000 × *g* to pellet cells membranes. Membranes were collected and solubilized in 1.25% DDM (n‐Dodecyl‐β‐D‐Maltopyranoside, Anatrace), gently shaken at room temperature for 2 hr, followed by ultracentrifugation at 142,000 × *g* for 30 min to remove insoluble materials.

Soluble membranes were loaded on 5 ml StrepTrap column (GE Healthcare), followed by extensive wash in LPA buffer and 0.05% DDM, and eluted in LPA buffer with 0.05% DDM and 2.5 mM desthiobiotin (Sigma). Eluted fractions were analyzed by SDS–PAGE, pulled, concentrated, and loaded on SEC column Superose 6 (GE Healthcare). Peak fractions were pulled, concentrated, and protein concentration was determined by OD_280_ measurement. To remove DDM, the concentrated complex solution was incubated with Amphipol A8‐35 (Anatrace) at 1:5 ratio for 4 hr, followed by overnight incubation with biobeads (Biorad). The sample was then reloaded on the Superose 6 column, and peak fractions were collected and concentrated for cryo‐EM studies.

### 4.3 Mass spectrometry preparation

Samples from purified T4CC mutants were run on SDS–PAGE, and bands corresponding to circa 20–35 kDa were sent for mass spec analysis using trypsin digestion. All MS/MS samples were analyzed using Sequest (Thermo Fisher Scientific, San Jose, CA, USA; version 27, rev. 12). Sequest was set up to search the uniprot‐*Legionella_pneumophila* database assuming trypsin digestion.

### 4.4 Western Blot analysis

To assess T4CC components stability, 48 hr heavy patch isogenic mutant strains (Δ*dotY*, Δ*dotZ*, Δ*dotY*/Z) expressing DotL_strep_, Lp01 expressing DoL_Strep_, and two control strains, Lp01 and Lp01_ΔT4SS_, were harvested and standardized to OD = 3.0 in water. Samples were added with SDS–PAGE loading buffer, and 10 µl samples were loaded on an SDS gel. DotM and IcmS were detected by anti‐DotM and anti‐IcmS antibodies, respectively, a kind gift from Joe Vogel. DotL was assessed using Strep•Tag^®^ II Antibody HRP Conjugate (Merk). All WB analyses were repeated three times in independent experiments.

### 4.5 Cell culture

*Acanthamoeba* *castellanii* (ATCC 30234) were cultured routinely at room temperature in ATCC medium 712 (PYG).

### 4.6 *Legionella* intracellular growth in eukaryotic hosts

Intracellular growth assays were performed as previously described (Meir et al., 2020). Briefly, *A. castellanii* cells were plated at 2 × 10^5^ cells/well and incubated at 37°C 2 hr prior infection. Two‐day heavy patch bacterial strains (Lp01 WT, GFP‐*dotY*, GFP‐*dotZ*) were grown on CYE plates with appropriate antibiotics (100 μg/ml streptomycin for WT and mutant strains, supplemented with 10 μg/ml chloramphenicol for the strains containing the complementing plasmids). Bacterial strains were added to *A. castellanii* plates at MOI of 0.1 (2 × 10^4^ cells per well, in AC medium) followed by centrifugation for 5 min at 350 × *g* at room temperature and incubation at 37°C for 1 hr.

### 4.7 DotY, DotZ, and T4CC cell localization

Imaging of *L. pneumophila* expressing Dot/Icm fluorescent fusions was carried out as previously described (Chetrit et al., 2018). Briefly, 2 day heavy patches were suspended in water, after which they were spotted on a thin pad of 1% agarose, covered with a cover slip and immediately imaged at room temperature. Fluorescence micrographs were captured using a Nikon Eclipse TE2000‐S inverted microscope equipped with a Spectra X light engine from Lumencor, CoolSNAP EZ 20 MHz digital monochrome camera from Photometrics and a Nikon Plan Apo100x objective lens (1.4 numerical aperture) under the control of SlideBook 6.0 (Intelligent Imaging Innovations). Samples were imaged using a 196 mW 485 nm LED light, with typical exposure times of 500–1,000 ms and 2 × 2 binning. sfGFP‐DotZ and its derivative strains were exposed for 5,000 ms. Polarity scores were calculated by measuring the ratio between the variance and the mean of the fluorescence signal at region of interest located between the cell poles.

### 4.8 Sequence conservation

Sequences for each protein of the T4CC from 39 *Legionella* species were found using BLASTP (Altschul et al., 1990) and aligned using ClustalOmega (Sievers et al., 2011) with default parameters. Then, ConSurf (Ashkenazy et al., 2016) and UCSF CHIMERA v1.13.1 (Pettersen et al., 2004) were used to visualize the conservation in sequence within the structure.

### 4.9 Cryo‐EM grid preparation and data acquisition

Aliquots of the purified T4CC_ΔDotYZ_ complex were applied to negatively glow discharged 300 mesh C‐flat 1.2/1.3 grids (Protochips, USA) and vitrified in liquid ethane using a Vitrobot Mark IV (Thermo Fisher, USA) at 4°C and 94% humidity. The data were collected at the eBIC National facility (Diamond Light Source, UK) operated at 300 keV and equipped with a Quantum energy filter. The images were collected with a post‐GIF K2 Summit direct electron detector operating in counting mode, at a magnification of 58,139, corresponding to a pixel size of 0.86 Å. An energy slit with a width of 20 eV was used during data collection. The dose rate on the specimen was set to 1.8834 e per Å^2^ per frame, and a total dose of 50.8 e per Å^2^ was fractionated over 27 frames. Data were collected using the EPU software (Thermo Fisher, USA) with a nominal defocus range set from −1.5 to −3.5 μm. A total of 2,203 micrographs were collected.

### 4.10 Cryo‐EM data processing

#### 4.10.1 Reprocessed T4CC_WT_ map with improved DotY density

The stack of T4CC_WT_ particles selected in our previous study (Meir et al., 2020) was subjected to 3D classification with a mask on DotY without image alignment using Tau = 20 using RELION 3.0 (Zivanov et al., 2018). The best resulting class corresponding to 183,397 particles was selected and imported to CRYOSPARC v2.9.0 (Punjani et al., 2017) to perform a 3D Refinement that resulted in an electron density map with an average resolution of 3.61 Å, with resolution extending locally to 4 Å for the DotY_middle_ domain, as estimated using gold standard Fourier shell correlation (FSC) with a 0.143 threshold. This map was AutoSharpen using PHENIX v1.14 (Adams et al., 2010) (Table S3).

#### 4.10.2 T4CC_WTminusYZ_ map

The data set of T4CC_WT_ particles collected in our previous study (Meir et al., 2020) was heterogenous, containing particles without DotY and DotZ. Thus, we used 3D and 2D classification with CryoSPARC v0.6.5 (Punjani et al., 2017) to select 330,583 of these DotY/Z‐less particles. This set was next refined in RELION 3.0 and subjected to 3D classification into eight classes using a mask focused on the DotLMN core, without image alignment using Tau = 20. The best class corresponding to 194,899 particles was selected. To limit anisotropy and improve the quality of the map, ~30,000 particles corresponding to preferential views were removed from the star files using rlnMaxValueProbDistribution criteria. The final subset of 166,260 particles was imported to CRYOSPARC v2.9.0, to perform 3D Refinement that resulted in an electron density map with a nominal resolution of 6.3 Å as estimated using gold standard FSC with a 0.143 threshold. This map was AutoSharpen using PHENIX v1.14 (Adams et al., 2010) (Table S3).

#### 4.10.3 T4CC_ΔDotYZ_ map

To validate the T4CC_WTminusYZ_ map described above, a small dataset of the T4CC_ΔDotYZ_ complex (purified from the Δ*dotYZ* strain) was collected and processed. RELION 3.0 was used for motion correction, and dose weighting with MOTIONCOR2 (Zheng et al., 2017) followed by CTF estimation using CTFFIND v4.1. 2D (Rohou & Grigorieff, 2015) projections templates, generated using the previously determined T4CC_WT‐YZ_ map, were used for particles picking with GAUTOMATCH v0.56 (Zhang, 2017). Dataset was subjected to multiple rounds of 2D classification with CRYOSPARC v0.6.5 (Punjani et al., 2017) leading to the selection of 236,653 out 657,783 particles. Further 3D heterogeneous classification resulted in the selection of 50,210 particles and 3D Refinement of these selected particles yielded an electron density map at 15 Å resolution as estimated using gold standard FSC with a 0.143 threshold (Table S3).

#### 4.10.4 IcmSW motions

Further image processing was performed to resolve maps with the IcmSW module at different positions relatively to the DotLMN core using the DotY/Z‐less particles described above (see the “T4CC_WTminusYZ_ map” section). The workflow used here was previously described to characterize the motions of IcmSW in T4CC_WT_ (Meir et al., 2020). In summary, multiple maps with IcmSW at different positions were obtained by doing iterative CRYOSPARC ab‐initio classification with the high‐resolution limited to 20 Å. In total, 43 maps for DotLMNYZ and 27 maps for DotLMN was obtained.

#### 4.10.5 IcmSW search volume analysis

UCSF CHIMERA v1.13.1 was used to generate the IcmSW search volume (see definition of search volume in main text). First, all maps with IcmSW density were manually superimposed onto the high resolution T4CC_WT_ map and saved using the command line “vop resample #1 OnGrid #0.” Next, a summation of all maps was generated using the command line “vop add #1‐43” (maps from T4CC_WT_) or “vop add #1‐27” (maps from T4CC_WTminusYZ_). Then, the density corresponding to the DotLMNYZ or DotLMN core was removed from the summation map, using the command line “vop subtract #44 #0” where #44 is the summation map and #0 is the high resolution core map. Remaining core densities were removed using the Volume Eraser tool. Finally, a filter was applied using Volume Filter tool with Filter type = Gaussian and a Width value = 2. The volume and area values were calculated using the Measure Volume and Area tool in Chimera (T4CC_WTminusYZ_: Sigma = 5.2 Volume = 387.1 Å^3^ Area = 31.31 Å^2^ | T4CC_WT_: Sigma = 12 Volume = 433.7 Å^3^ Area = 31.55 Å^2^).

### 4.11 DotY_middle_ model building

Side chain definition for DotY in the 3.61 Å “reprocessed T4CC_WT_” map of the region was good enough to build side chains for DotY_NTD_ (residues 1–77) and for the first α‐helix of DotY_middle_ and the loop after it (residue 78–106). The remaining density for DotY_middle_ (residues 107–192) was of a lesser resolution and thus only the backbone could be traced. All regions with side chains definition were built de novo in COOT v0.8.9.1 (Emsley & Cowtan, 2004) and the structure was refined using real‐space refinement in PHENIX v1.14. For the regions where only main chain definition was observed, the Cα backbone was fitted into the density map (COOT) starting with a model generated by I‐TASSER (Yang et al., 2015) and aided by secondary structure prediction by PSIPRED 4.0 (McGuffin et al., 2000). Finally, the resulting DotY model was refined using PHENIX v1.14 real‐space refinement and MOLPROBITY v4.4 was used to evaluate the quality of the structure (Table S3).

### 4.12 Figure generation

All figures were generated using CHIMERAX v0.91 (Pettersen et al., 2021).

## CONFLICT OF INTEREST

The authors declare no conflict of interest.

## AUTHOR CONTRIBUTIONS

AM cloned, expressed, and purified the T4CC and its variants and obtained the NS data. AM, MKH, and NL prepared the Cryo‐EM grids and NL collected EM data. KM performed the EM processing, model building, and analyzed the IcmSW motion zone. KM and AM performed the sequence conservation analysis. AM and DC generated the *Legionella* mutants and AM tested them. CR supervised the biological work. AM and GW supervised the biochemical work. GW supervised the structural work. AM, KM, and GW wrote the article.

## Supporting information

Supplementary Material

## Data Availability

The DotY structure has been deposited to the PDB together with the map that was used to generate it (EMDB and PDB codes 13083 and 7OVB, respectively). EM maps T4CC_WTminusYZ_ and T4CC_ΔdotYZ_ have also been deposited (EMD‐13858 and EMD‐13859, respectively). Any supplementary data generated during the current study are available from the corresponding author on request.
