# Supplementary material for: Proteins DotY and DotZ modulate the dynamics and localization of the type IVB coupling complex of Legionella pneumophila
Source: Mol Microbiol. 2021 Dec 6;117(2):307–19. doi: 10.1111/mmi.14847 (PMC9300119; doi:10.1111/mmi.14847)
Supplement: Supplementary file 1 — Supplementary Material [file MMI-117-307-s001.pdf]

SUPPLEMENTARY INFORMATION

The proteins DotY and DotZ modulate the dynamics and localization of the type IVB coupling complex of *Legionella pneumophila*

Kevin Macé[1,4], Amit Meir[1,2,4*], Natalya Lukoyanova[1], Luying Liu[2], David Chetrit[2], Manuela K Hospenthal[1,5], Craig R. Roy[2*], and Gabriel Waksman[1,3*]

[1] Institute of Structural and Molecular Biology, Birkbeck and UCL, Malet Street, London WC1E 7HX, UK; [2] Boyer Center for Molecular Medicine, Department of Microbial Pathogenesis, Yale University, 295 Congress Avenue, New Haven, CT 06536-0812, USA; [3]Institute of Structural and Molecular Biology, University College London, Gower Street, London WC1E 6BT, UK. [4] These authors contributed equally to this work. [5]Present address: Institute of Molecular Biology and Biophysics, Department of Biology, ETH Zürich, Otto-Stern-Weg 5, 8093 Zürich, Switzerland

* Author for correspondence:

a.meir@mail.cryst.bbk.ac.uk, craig.roy@yale.edu and g.waksman@mail.cryst.bbk.ac.uk

SUPPLEMENTARY TABLES

Table S1: Strains, primers and plasmids used in this study

A. Strains

Strain	Description	Source/ Reference
***E.coli K12* strains used in this study**		
E. coli Mach1	Δ*recA1398 endA1 fhuA* Φ80Δ(*lac*)M15 *Δ(*lac*)X74* hsdR*(*$r_K^- m_K^+$*)*	Invitrogen
E. coli DH5α λpir	sup E44, ΔlacU169 (ΦlacZΔM15), recA1, endA1, hsdR17, thi-1, gyrA96, relA1, λpir phage lysogen	(Zuckman et al., 1999)
E. coli CR019	MT607 *E. coli* containing plasmid pRK600; ColE1 replicon with RK2 transfer genes, Cm^R	(Hubber et al., 2014)
***Legionella pneumophila* strains used in this study**		
Lp01	$Strep^R$, *Legionella pneumophila* serogroup 1, Lp01 *rpsL*	(Berger and Isberg, 1993)
Lp02	$Strep^R$, *Legionella pneumophila* serogroup 1, Lp01 *rpsL SmR, r–thyA*	(Berger and Isberg, 1993)
Lp01 $DotL_{strep}$	$Strep^R$, Lp01 with a Strep tag at the C-terminus of DotL	This study
Lp02 $DotL_{strep}$	$Strep^R$, Lp02 with a Strep tag at the C-terminus of DotL	This study
ΔT4SS	$Strep^R$, Lp01 chromosomal deletions of three loci: *icmX–dotA*, *dotB–dotD*, and *icmT–dotU*	(Kubori et al., 2014)
Lp01 L_{strep} Δ*dotY*	$Strep^R$, Lp01 chromosomal deletions of *dotY* with a Strep tag at the C-terminus of DotL	This study
Lp01 L_{strep} Δ*dotZ*	$Strep^R$, Lp01 chromosomal deletions of *dotZ* with a Strep tag at the C-terminus of DotL	This study
Lp01 L_{strep} Δd*otYdotZ*	$Strep^R$, Lp01 chromosomal deletions of *dotY* and *dotZ* with a Strep tag at the C-terminus of DotL	This study
Lp02 L_{strep} Δ*dotY*	$Strep^R$, Lp02 chromosomal deletions of *dotY* with a Strep tag at the C-terminus of DotL	This study
Lp02 L_{strep} Δ*dotZ*	$Strep^R$, Lp02 chromosomal deletions of *dotZ* with a Strep tag at the C-terminus of DotL	This study
Lp02 L_{strep} Δ*dotYdotZ*	$Strep^R$, Lp02 chromosomal deletions of *dotY* and *dotZ* with a Strep tag at the C-terminus of DotL	This study
Lp01 DotL sfGFP	$Strep^R$, Lp01 with sfGFP at the C-terminus of DotL	(Chetrit et al., 2018)
Δ*dotY* DotL sfGFP	$Strep^R$, Lp01 *dotY* chromosomal deletion with sfGFP at the C-terminus of DotL	This study
Δ*dotZ* DotL sfGFP	$Strep^R$, Lp01 *dotZ* chromosomal deletion with sfGFP at the C-terminus of DotL	This study
Δ*dotYdotZ* DotL sfGFP	$Strep^R$, Lp01 *dotY* and *dotZ* chromosomal deletion with sfGFP at the C-terminus of DotL	This study
Δ*dotY* DotL sfGFP +pDotY	$Strep^R$, CM^R, Δ*dotY* DotL sfGFP + pDotY	This study

Δ*dotZ* DotL sfGFP + p*dotZ*	Strep^R, CM^R, Δ*dotZ* DotL sfGFP + p*dotZ*	This study
Δ*dotYdotZ* DotL sfGFP +p*dotY*	Strep^R, CM^R, Δ*dotYZ* DotL sfGFP + p*dotY*	This study
Lp01 sfGFP DotY	Strep^R, Lp01 with SFGFP at the N-terminus of DotY	This study
Lp01 sfGFP DotY	Strep^R, Lp01 with SFGFP at the N-terminus of DotY	This study
Lp01 sfGFP DotY Δ*dotZ*	Strep^R, Lp01 dotZ chromosomal deletion with sfGFP at the N-terminus of DotY	This study
Δ*dotB*	Strep^R, Lp01 *dotB* chromosomal deletion	(Meir et al., 2020)
ΔBL	Strep^R, Lp01 *dotB* chromosomal deletion followed by *dotL* chromosomal deletion	This study
Lp01 sfGFP DotY ΔBL	Strep^R, Lp01 Δ*dot*B Δ*dot*L with sfGFP at the N-terminus of DotY	This study
Lp01 sfGFP DotZ	Strep^R, Lp01 with sfGFP at the N-terminus of DotZ	This study
Lp01 sfGFP DotZ	Strep^R, Lp01 with sfGFP at the N-terminus of DotZ	This study
Lp01 sfGFP DotZ Δ*dotY*	Strep^R, Lp01 dotY chromosomal deletion with sfGFP at the N-terminus of DotZ	This study
Lp01 sfGFP DotZ ΔBL	Strep^R, Lp01 ΔBL with sfGFP at the N-terminus of DotZ	This study
**Eukaryotic cell lines used in this study**		
Acanthamoeba castellanii	ATCC 30234	

B. Plasmids

Construct	Description and Reference	Primers Forward	Primers Reverse
pSR47S	Km^R; (Merriam et al., 1997))		
pSR47S-Lin	Linearize pSR47S with In-Fusion to yield an open vector		
pSR47S-YI	Km^R; Full length DotY (lpg0294) from *L. pneumophila* with 1000bp upstream and downstream cloned into pSR47S-Lin backbone (Meir et al., 2020))		
pSR47S-ZI	Km^R; Full length DotZ (lpg1549) from *L. pneumophila* with 1000bp upstream and downstream cloned into pSR47S-Lin backbone (Meir et al., 2020))		
pSR47S-LI_{strep}	Km^R; pSR47S-LI derivative encoding a strep tag on the C-terminal of DotL (Meir et al., 2020))		
pSR47S-Δ*dotY*	Km^R; pSR47S-YI derivative with *dotY* deletion (Meir et al., 2020))		
pSR47S-Δ*dotZ*	Km^R; pSR47S-ZI derivative with *dotZ* deletion (Meir et al., 2020))		
pSR47S-YILin	Linearize pSR47S-YI with In-Fusion to yield an open vector (this study)	p47031F	p47032R

pSR47S-sfGFPYI	Km^R; pSR47S-YI derivative encoding sfGFP fusion at the N-terminal of DotY (this study)	sfGFP03F	sfGFP04R
pSR47S-ZILin	Linearize pSR47S-ZI with In-Fusion to yield an open vector (this study)	p47027F	p47028R
pSR47S-SFGFPZI	Km^R; pSR47S-ZI derivative encoding sfGFP fusion at the N-terminal of DotZ (this study)	sfGFP03F	sfGFP04R
pJB1806	Cm^R Amp^R; (Bardill et al., 2005))		
pJB1806-Lin	pJB1806 without its lac promoter, linearized with In-Fusion to yield an open vector (Meir et al., 2020))		
p*dotY*	Cm^R; *dotY* with 200bp upstream and downstream cloned into pJB1806-Lin backbone (Meir et al., 2020))		
p*dotZ*	Cm^R; *dotZ* with 200bp upstream and downstream cloned into pJB1806-Lin backbone (Meir et al., 2020))		
pSR47S-Δ*dotL*	Km^R; pSR47S-LI derivative with *dotL* deletion (Chetrit et al., 2018))		
pDotB	Cm^R;DotB with C-terminus Strep tag cloned into pMMB207-Lin (Meir et al., 2020))		

C. Primers

**Primer name**	**Primer sequence 5'-3'**
p47027F	aaggtgctggcggatccagcagcggcggtggcgcgatggacgagatcaaaaaagatgatgaattgagtc
p47028R	ggaacaacaccggtgaacagttcttcacctttagacatttttatccaatttaaattctttaaagtatgtgatataaggg
p47031F	aaggtgctggcggatccagcagcggcggtggcgcgatgccaaaatacacactgcccacaagag
p47032R	ggaacaacaccggtgaacagttcttcacctttagacataacttactctcaacaatgtatatttggtccagg
sfGFP03F	atgtctaaaggtgaagaactgttcaccggtg
sfGFP04R	cgcgccaccgccgctgctggatccgccagcacctttgtagag

Table S2. Mass spectrometry analysis of T4CC proteins in isolated complex sample**.** T4CC proteins were fully identified by mass spec as previously described in Meir et al 2020 (Meir et al., 2020). WT and mutants' identification was conducted in a different MS facility, hence the different value range. Scaffold (version Scaffold_4.10.0, Proteome Software Inc., Portland, OR) was used to validate MS/MS based peptide and protein identifications. Peptide identifications were accepted if they could be established at greater than 80.0% probability by the Peptide Prophet algorithm (Keller et al., 2002) with Scaffold delta-mass correction. Protein identifications were accepted if they could be established at greater than 99.0% probability and contained at least 2 identified peptides. Protein probabilities were assigned by the Protein Prophet algorithm (Nesvizhskii et al., 2003). Proteins that contained similar peptides and could not be differentiated based on MS/MS analysis alone were grouped to satisfy the principles of parsimony. The data were searched against *L. pneumophila* UniProt protein database.

Strain	Identified Protein	Accession Number	Molecular Weight	Quantitative Value (Normalized Total Spectra)
WT	DotZ	Q5ZV91_LEGPH	34 kDa	2009
	DotY	Q5ZYR7_LEGPH	26 kDa	1698
	LvgA	Q5ZY48_LEGPH	23 kDa	210
	DotN	Q5ZYB7_LEGPH	24 kDa	159
Δ*dotY*	LvgA	Q5ZY48_LEGPH	23 kDa	30.13
	DotN	Q5ZYB7_LEGPH	24 kDa	16.28
Δ*dotZ*	DotN	Q5ZYB7_LEGPH	24 kDa	45.47
	LvgA	Q5ZY48_LEGPH	23 kDa	44.38

Table S3. Cryo-EM data collection, refinement and validation statistics.

Complex/Subcomplex	Reprocessed T4CC$_{WT}$	T4CC$_{WTminusYZ}$ at 6.3 Å	T4CC$_{\Delta DotYZ}$ at 15 Å
PDB	7OVB	-	-
EMDB	13083	13858	13859
Data collection and processing			
Magnification	130,000	130,000	130,000
Voltage (kV)	300	300	300
Electron exposure (e^{-}/Å^2)	54	54	50.8
Defocus range (μm)	-1.5 to -3.5	-1.5 to -3.5	-1.5 to -3.5
Pixel size (Å)	1.045	1.045	1.045
Symmetry imposed	C1	C1	C1
Initial particle images (no.)	626,230	626,230	236,653
Final particle images (no.)	183,397	166,260	50,210
Map resolution (Å)	3.61	6.30	15
FSC threshold	0.143	0.143	0.143
Map resolution range (Å)	3.4-8	5.5-8	12-20
Map sharpening B factor (Å^2)	-43.12	-187.45	-
Refinement[1]			
Initial model used (PDB code)	6sz9		
Model resolution (Å)	3.5		
FSC threshold	0.143		
CC Model vs. Data (mask)	0.81		
Model composition			
Nonhydrogen atoms	10854		
Protein residues	1405		
Ligands	1		
B factors (Å^2)			
Protein	80.96 (35 -185)		
Ligand	70.19		
R.m.s. deviations			
Bond lengths (Å)	0.013		
Bond angles (°)	1.113		
Validation[1]			
MolProbity score	2.42		
Clashscore	21.40		
Rotamer outliers (%)	0.00		
Ramachandran plot			
Favored (%)	88.32		
Allowed (%)	11.68		
Disallowed (%)	0.00		

[1.] Determined by phenix.mtriage and phenix.molprobity (values in parenthesis denote highest and lowest values).

SUPPLEMENTARY FIGURES

Figure S1. Original gels from which Figure 2b was extracted. Legend is as in Figure 2b.

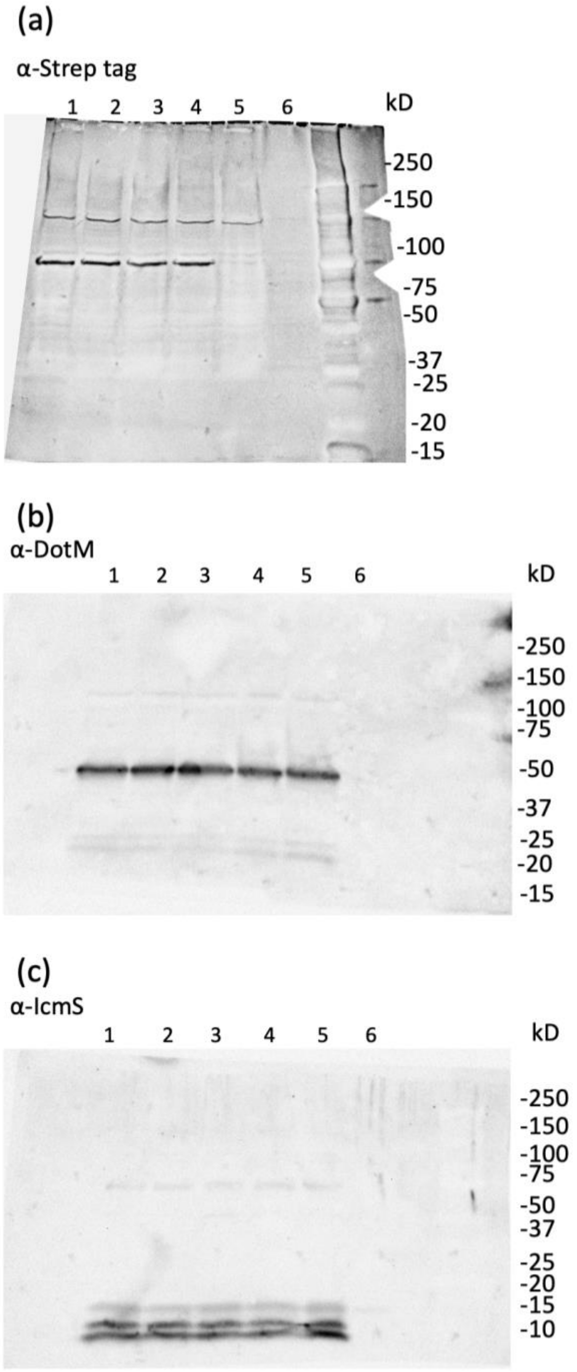

Figure S2. sfGFP-DotY and sfGFPDotZ intracellular growth**.** Intracellular growth of sfGFP-*dotY* and sfGFP-*dotZ* in *A. castellanii*. Wild-type (Lp01, filled circles), sfGFP-*dotY* (empty triangles) and sfGFP-*dotZ* (empty squares). Data are representative of biological triplicates. Graphs reports mean intracellular growth fold ± standard deviation for each strain. P values of sfGFP strains in comparison to wild-type were calculated by two-tailed Student's t test, and P>0.05, i.e. not significant. Lp01 *dotLsfGFP* intracellular growth has been previous described (Chetrit et al 2018) to have no significance difference comparing to Lp01 WT.

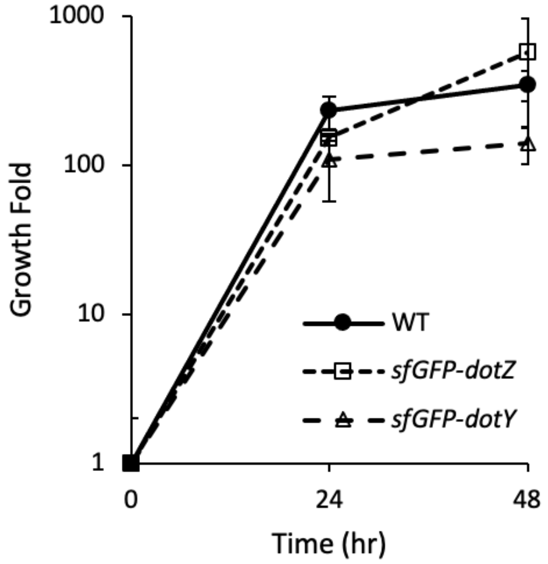

REFERENCES FOR SUPPLEMENTARY INFORMATION

BARDILL, J. P., MILLER, J. L. & VOGEL, J. P. 2005. IcmS-dependent translocation of SdeA into macrophages by the Legionella pneumophila type IV secretion system. *Mol Microbiol,* 56, 90-103.

BERGER, K. H. & ISBERG, R. R. 1993. Two distinct defects in intracellular growth complemented by a single genetic locus in Legionella pneumophila. *Mol Microbiol,* 7, 7-19.

CHETRIT, D., HU, B., CHRISTIE, P. J., ROY, C. R. & LIU, J. 2018. A unique cytoplasmic ATPase complex defines the Legionella pneumophila type IV secretion channel. *Nat Microbiol,* 3, 678-686.

HUBBER, A., ARASAKI, K., NAKATSU, F., HARDIMAN, C., LAMBRIGHT, D., DE CAMILLI, P., NAGAI, H. & ROY, C. R. 2014. The machinery at endoplasmic reticulum-plasma membrane contact sites contributes to spatial regulation of multiple Legionella effector proteins. *PLoS Pathog,* 10, e1004222.

KELLER, A., NESVIZHSKII, A. I., KOLKER, E. & AEBERSOLD, R. 2002. Empirical statistical model to estimate the accuracy of peptide identifications made by MS/MS and database search. *Anal Chem,* 74, 5383-92.

KUBORI, T., KOIKE, M., BUI, X. T., HIGAKI, S., AIZAWA, S. & NAGAI, H. 2014. Native structure of a type IV secretion system core complex essential for Legionella pathogenesis. *Proc Natl Acad Sci U S A,* 111, 11804-9.

MEIR, A., MACE, K., LUKOYANOVA, N., CHETRIT, D., HOSPENTHAL, M. K., REDZEJ, A., ROY, C. & WAKSMAN, G. 2020. Mechanism of effector capture and delivery by the type IV secretion system from Legionella pneumophila. *Nat Commun,* 11, 2864.

MERRIAM, J. J., MATHUR, R., MAXFIELD-BOUMIL, R. & ISBERG, R. R. 1997. Analysis of the Legionella pneumophila fliI gene: intracellular growth of a defined mutant defective for flagellum biosynthesis. *Infect Immun,* 65, 2497-501.

NESVIZHSKII, A. I., KELLER, A., KOLKER, E. & AEBERSOLD, R. 2003. A statistical model for identifying proteins by tandem mass spectrometry. *Anal Chem,* 75, 4646-58.

ZUCKMAN, D. M., HUNG, J. B. & ROY, C. R. 1999. Pore-forming activity is not sufficient for Legionella pneumophila phagosome trafficking and intracellular growth. *Mol Microbiol,* 32, 990-1001.
